# Sports Nutrition and Food Knowledge among Malaysian University Athletes

**DOI:** 10.3390/nu14030572

**Published:** 2022-01-28

**Authors:** Nur Syazana Nor Azizam, Siti Nurhazlin Yusof, Jonie Jerypin Amon, Azimah Ahmad, Nik Shanita Safii, Nor Aini Jamil

**Affiliations:** 1 Centre for Community Health Studies (ReaCH), Faculty of Health Sciences, Universiti Kebangsaan Malaysia, Kuala Lumpur 50300, Malaysia; a167673@siswa.ukm.edu.my (N.S.N.A.); a168797@siswa.ukm.edu.my (S.N.Y.); a168639@siswa.ukm.edu.my (J.J.A.); nikshanita@ukm.edu.my (N.S.S.); 2 Faculty of Medicine and Defence Health, National Defence University of Malaysia, Kuala Lumpur 57000, Malaysia; azimah@upnm.edu.my

**Keywords:** sports nutrition, food knowledge, university athletes, nutrition education

## Abstract

Sports nutrition and food knowledge can influence athletes’ dietary intake, potentially affecting athletic performance. Limited studies have been conducted to identify sports nutrition and food knowledge among Malaysian university athletes. This study aimed to determine Malaysian university athletes’ knowledge of sports nutrition and food, and their reference sources and preferred sports nutrition education programme. Seventy athletes (52.9% females, mean weight = 61.8 kg; height = 1.66 m) aged 18.5–22.4 years responded to an online survey administered using a Google Form. A score of ≥60% was considered as adequate knowledge. The average overall knowledge score was 58.6%. The highest knowledge score was for sports nutrition, specifically food intake periodicity (84.8%), while the lowest was for general food knowledge on fat (44.6%). An analysis of specific questions revealed a lack of understanding about the recommended daily intake of fruits and vegetables (only 14.3% answered correctly), the roles of vitamins and minerals in energy production (21.4%), and good sources of unsaturated fat (37.2%). The academician was the primary reference source (81%), while magazines were the least referred to sources (36%). The university athletes preferred sports camps (33%) over the other nutrition education programme options. More initiatives are needed to improve Malaysian university athletes’ understanding and knowledge of sports nutrition and general food.

## 1. Introduction

Athletes’ nutritional requirements are primarily determined by the demands of their activities and goals to achieve peak sports performance and overall health [1,2]. Appropriate nutritional practice is essential since it influences almost every process in the body, from energy production to the recovery period following exercise [3]. In addition, individual dietary intakes may be influenced by knowledge, attitude, and nutrition-related information resources available for obtaining nutrition-related information [4].

Nutritional knowledge is a modifiable factor influencing dietary behaviours. Athletes’ understanding of sports nutrition can thus influence their food choices, which in turn can affect their overall athletic performance [5,6]. Hence, athletes should obtain appropriate nutritional information from a credible and certified source, as this will shape their understanding of nutrition [7,8]. One strategy to increase nutritional awareness in athletes is through nutrition education programmes [9,10], such as individual nutrition counselling, group activities workshops, or online educational materials [11,12,13]. Sports camps are a common type of athlete training that is organized by an institution to prepare athletes for competition [14]. Nutritional education sessions can be incorporated into sports camp activities.

Only a few studies have been conducted in Malaysia to determine nutrition knowledge among athletes, specifically on general nutrition [15], hydration [16], and sports supplements [17]. So far, no studies have assessed Malaysian athletes’ overall sports nutrition knowledge. Such information is important since it may influence their practice and behaviour, which can affect their sports performance and general health. Several studies, however, observed that knowledge level does not always influence sports nutrition practices [16,18]. For instance, Sedek et al. (2015) found no association between Malaysian university athletes’ knowledge of hydration and fluid replacement (mean score = 74.1%) and their practices (60.9%), but a moderate relationship between attitudes and practices [16]. In another study conducted among university sports teams and local clubs in Ireland, nutritional knowledge was higher among euhydrated athletes at the start of exercise (55.2%) than among dehydrated athletes (50.6%), but the overall nutritional knowledge score was still low (52.9%) [18]. These findings suggest that more research is needed to explore other factors that influence athletes’ knowledge and practice, and that knowledge levels vary by population and sociodemographic profiles. This study aimed to assess university athletes’ sports nutrition and food knowledge based on sociodemographic profiles and to identify their reference sources and preferred medium of sports nutrition education programmes.

## 2. Materials and Methods

This study was a cross-sectional study among athletes with a sport science backgrounds in a university in Kuala Lumpur, Malaysia, using a purposive sampling technique. We included athletes aged 18 years and above who were citizens of Malaysia and excluded those with chronic illnesses such as cardiovascular disease, hypertension, or diabetes mellitus. Ethical approval was obtained from the University’s Research Ethics Committee (reference code: UKM PPI/111/8/JEP-2021-227), and all participants gave written informed consent before the start of the study.

The online survey was carried out using Google Form, and the link to the survey was distributed to participants via the WhatsApp application. The data collected include sociodemographic profiles such as sex, age, level of education, sports participation, body weight, and height. Participants were also asked about their previous participation in a nutrition education program or consultation sessions with a nutritionist or dietitian, their reference sources for knowledge on sports nutrition, and their preferred medium of a nutritional educational program.

Sports nutrition and food knowledge were measured using a previously validated questionnaire, the nutritional knowledge for young and adult athletes (NUKYA) [4]. This questionnaire was developed based on the latest athletes’ dietary guidelines and was thoroughly reviewed by experts. We modified some of the questions to ensure that they were relevant to the local practices and culture. The final questionnaire is composed of 24 questions with 57 items covering four different sections: macronutrient (27 items), micronutrient (19 items), hydration (8 items) and food intake periodicity (3 items). We gave one point for each correct answer and zero points for an incorrect answer or “I do not know.” The total raw score (ranged from 0 to 57) was proportionately transformed to a scale of 0–100. Due to the absence of a scoring system [4], we classified scores of 60% and above as adequate knowledge, and vice versa [8].

All analyses were performed using the IBM SPSS Statistics software (version 25.0, IBM Corp., Armonk, NY, USA). Data were checked for normality using the Shapiro–Wilk test, a histogram and scatterplot. Descriptive data were reported as the mean and standard deviation for continuous data, and frequency and percentage for categorical data. An independent *t*-test and ANOVA were used to compare athletes’ sports nutrition and food knowledge based on sociodemographic factors. A *p*-value < 0.05 was considered a statistically significant.

## 3. Results

A total of 70 university athletes (53% females) aged between 18 and 22 years participated in this study (Table 1). The majority of the participants were Malay (96%) and normal weight status (80%). Most athletes were degree students (60%) and first-year students (69%). Almost half of the participants participated in a team sport (43%) and competed at the state level (46%). Most participants (96%) had taken a nutrition course, and half had consulted with a dietitian or nutritionist.

The average overall sports nutrition and food knowledge score was 58.6%, ranging from 28% to 81% (Table 2). The highest knowledge score was for the food intake periodicity (mean score = 84.8%), with 95.7% demonstrating adequate knowledge in this section. Meanwhile, the fat section had the lowest knowledge score (mean score = 44.6%), with only 22.9% having adequate knowledge about fat.

Further analysis of the specific question and answer revealed that most participants could not identify the recommended amount of fruit and vegetable intake (only 14.3% answered correctly) and the roles of vitamins and minerals in energy production (21.4%) (Table 3). Only dietary protein sources achieved an adequate knowledge score (mean score = 71.2%), while scored for other nutrients, especially unsaturated fat, were low (37.2%). For sports nutrition knowledge, questions related to hydration, such as suitable drinks during exercise and strategies for re-hydration, required further attention for nutrition education.

Based on sociodemographic profiles, the university athletes studying a degree (mean knowledge score = 61.8%) had greater knowledge than the diploma students (53.8%, *p* = 0.001) (Table 4). Those who had previously consulted with a dietitian/nutritionist had a significantly lower mean knowledge score (55.6%) than their counterparts (61.5%, *p* = 0.02). There was no significant difference in the mean knowledge score for other sociodemographic profiles.

Table 5 shows the reference source for sports nutrition information among university athletes. The top four resources were academicians (81%), internet (63%), medical officers (57%), and dietitians/nutritionists (54.3%). Meanwhile, the most preferred medium for nutrition education programs was sports camps (33%), followed by videos (29%), talks (24%), and brochures/posters (14%) (Table 6).

## 4. Discussion

This study assessed sports nutrition and food knowledge among Malaysian university athletes, including knowledge on macronutrient, micronutrient, hydration, and food intake periodicity. Overall, the athletes had inadequate sports nutrition and food knowledge (mean score = 58.6%), with more than half having a knowledge score <60%. This finding was consistent with previous studies conducted among university athletes in the United States (59.6%) [19] and basketball athletes in Lebanon (50.2%) [8]. In contrast, elite athletes from five universities in the Punjab reportedly had good sports nutrition knowledge (71.2%) [20].

The majority of the university athletes in the current study (96%) correctly answered the questions about food intake periodicity, especially the pre-exercise meal timing. In contrast, elite athletes from the Australia League Club scored 56.6% and 57.5% on pre and post-exercise sports nutrition questions, respectively [21]. Adequate knowledge of protein food sources and its function was also demonstrated in the current study, implying that nutrition education in this area could be effective. However, the athletes could not distinguish between low and high unsaturated fat-food sources, similar to previous studies among football players, suggesting that nutrition education about fat received less emphasis than nutrition education for other macronutrients [21,22,23]. Furthermore, most athletes in our study (86%) could not correctly state the recommended daily intake of fruits and vegetables, consistent with a previous study [21].

We found a significant difference in knowledge scores based on education level, in which the degree educated athletes had a higher mean score (61.8%) than the diploma university athletes (53.8%). Previous studies have also shown that education influences sports nutrition knowledge [24,25]. Further analysis in our study revealed that all degree athletes had taken a nutrition course, compared to only 89.3% among diploma athletes. However, whether the nutrition course studied was part of their degree curriculum or an extra course is unknown. Future research should collate detailed information on this, including the course level. Surprisingly, our data showed that university athletes who had never seen a dietitian/nutritionist had a higher knowledge score (61.5%) than their counterparts (55.6%), contradicting previous findings [8]. Yet, we did not collect information on the consultation sessions’ frequency, duration, and purpose, which could explain this finding. In addition, the university does not currently have an in-house dietitian/nutritionist, implying that the university athletes receive dietary advice from other institutions.

Previous studies found significant differences in knowledge scores based on sex [5,8,26], BMI classification [26], and sports category [26], which we did not observe in this study. While the general principle of sports nutrition is universal for all athletes worldwide, different studies used different sets of questions and assessed different knowledge domains. Furthermore, no standard questionnaire for sports nutrition knowledge has been established [27]. We adapted a previously developed and validated questionnaire that focused on key topics in athlete general nutrition education [4]. However, this questionnaire does not include questions about supplement use or weight management, which highly depend on individual needs or sport type.

Most university athletes in the current study obtained their nutritional information from reputable personnel such as academicians, medical officers and dietitians/nutritionists, as well as online resources from the internet. The least reference source was magazines. Our findings differed from an earlier study in Malaysia in which they reported that the internet, magazines, family and friends, and television were the top sources of nutrition information for university athletes [15]. These findings showed that individuals’ preferences vary and are influenced by many factors, including social and environmental factors. Only some of the athletes in this study had access to a sports dietitian/nutritionist. The university sports center could devise a comprehensive program by collaborating with a sports dietitian/nutritionist so that all university athletes are able to receive nutritional information and apply it in their daily activities to improve their sports performance. This can be delivered through sports camps, which are preferred by current university athletes. Camp is known for providing opportunities for skill development and personal growth, as well as encouraging peer and instructor relationships [28].

Some limitations may affect the generalizability of these results. Our study was conducted during the COVID-19 pandemic using WhatsApp for recruitment and online survey through Google Form. We could not tell for certain whether they discussed the questions with family and friends or searched the answer online, although reminders were sent not to be concerned about the results. The study was also conducted in a university, thus does not represent all Malaysian university athletes.

## 5. Conclusions

Overall, sports nutrition and food knowledge among university athletes, in this study, were inadequate. This may predispose athletes to make poor nutrition choices due to misconceptions and misinformation, which may have a negative impact on their health and sports performance. Therefore, additional measures to improve sports nutrition and general food knowledge are required. Based on our study, university athletes preferred sports camps as a medium for nutrition education programs.

## Figures and Tables

**Table 1:** Sociodemographic profiles of the university athletes (*n* = 70).

	*n*	%	Mean Score (SD)	Min, Max
Age (years)			20.6 (1.0)	18.5, 22.4
Sex				
Male	33	47.1		
Female	37	52.9		
Ethnicity				
Malay	67	95.7		
Indian	3	4.3		
Body weight (kg)			61.8	44.0, 91.0
Height (m)			1.66	1.42, 1.83
BMI (kg/m^2^)			22.4	16.5, 31.9
BMI status				
Underweight	4	5.7		
Normal	56	80.0		
Overweight	8	11.4		
Obese	2	2.9		
Level of study				
Diploma	28	40.0		
Degree	42	60.0		
Year of study				
First-year	48	68.6		
Second-year	22	27.1		
Third-year	3	3.3		
Sport category				
Team sport	30	42.9		
Skills	14	20.0		
Athletic	11	15.7		
Combat sport	10	14.3		
Racket	5	7.1		
Level of sport participation				
University	14	20.0		
State	32	45.7		
National	17	24.3		
International	7	10.0		
Attended a nutrition course	67	95.7		
Consulted with a dietitian/nutritionist	35	50.0		

**Table 2:** Sports nutrition and food knowledge scores and proportion with adequate knowledge status.

Section	Mean Score (SD), %	Adequate Knowledge Status (Score ≥ 60%)	
		*n*	%
All section	58.6 (10.2)	31	44.3
Macronutrient	58.7 (13.1)	34	48.6
Carbohydrate	63.3 (13.8)	49	70.0
Protein	69.8 (21.8)	45	64.3
Fat	44.6 (24.3)	16	22.9
Micronutrient	54.4 (16.0)	25	35.7
Hydration	58.2 (17.1)	38	54.3
Food intake periodicity	84.8 (19.4)	67	95.7

**Table 3:** Responses to sports nutrition and food knowledge questions.

Section	Question/Scope	Athletes with Correct Answer	Mean Score
		*n* (%)	(%)
Macronutrient (carbohydrate)	1. Identifying good food sources of carbohydrates (9 items).		59.0
	2. Should an athlete who wants to lose weight completely eliminate carbohydrates from his/her diet?	54 (77.1)	
	3. Are carbohydrates stored in the muscle as glycogen?	61 (87.1)	
Macronutrient (protein)	4. Does the muscle use protein as the main energy source during exercise?	43 (61.4)	
	5. Identifying good food sources of protein (6 items).		71.2
Macronutrient (fat)	6. Do fats play an important role in the body?	61 (87.1)	
	7. Do saturated and unsaturated fats have the same impact on health?	38 (54.3)	
	8. Identifying foods with a high or low unsaturated fat content (7 items).		37.2
Micronutrient	9. How many servings of fruits and vegetables is recommended to eat per day?	10 (14.3)	
	10. Can the human body get most of its vitamin D from sunlight exposure?	22 (31.4)	
	11. Are vitamins and minerals a good source of energy?	15 (21.4)	
	12. Identifying good food sources of iron (8 items).		59.1
	13. Identifying good food sources of calcium (8 items).		61.6
Hydration	14. Your athletic performance will decrease if you lose 2% of your body weight (for example, 1.5 kg if you weigh about 75 kg) due to water loss.	47 (67.1)	
	15. To be well hydrated during sports training, you have to wait until you are thirsty to drink.	64 (91.4)	
	16. To fully rehydrate after exercise, you need to drink a volume of liquid greater than the volume of water lost during exercise (which we know by weighing yourself before and after training or competition).	63 (90.0)	
	17. Fruit juice is a liquid suitable to drink in the training session and in the middle of the match.	33 (47.1)	
	18. Energy drinks like “Red Bull” are recommended for athletes to ingest during exercise.	31 (44.3)	
	19. What do you think is the most suitable urine color before training?	32 (45.7)	
	20. During intense or prolonged exercise, what is the best way to replace the water that is lost in the form of sweat?	27 (38.6)	
	21. The percentage of carbohydrates in an isotonic sports drink.	29 (41.4)	
Food intake periodicity	22. What is the optimum time to eat and drink something for kick-starting recovery after exercise or competition?	59 (84.3)	
	23. The most important nutrient(s) to ingest after training.	23 (75.7)	
	24. Should the last main meal (breakfast, lunch or dinner) be eaten at least 3–4 h before a competition/ exercise?	66 (94.3)	

**Table 4:** Comparison of sports nutrition and food knowledge score based on sociodemographic profiles.

	Mean Score (SD)	*p*-Value
Sex		0.334
Male	57.3 (9.2)	
Female	59.7 (11.1)	
Body weight status		0.518
Normal	58.3 (10.3)	
Overweight/obese	60.5 (10.2)	
Level of study		0.001
Diploma	53.8 (9.1)	
Degree	61.8 (9.8)	
Year of study		0.444
First-year	59.2 (10.8)	
Second & third-year	57.2 (9.0)	
Sport category		0.196
Team sport	58.3 (9.6)	
Skills	64.0 (11.7)	
Athletic	54.6 (13.2)	
Combat sport	58.1 (5.5)	
Racket	55.4 (6.2)	
Level of sport participation		0.833
University	60.4 (9.2)	
State	57.8 (9.6)	
National	59.2 (11.0)	
International	56.9 (14.1)	
History of attending a nutrition course		0.908
Yes	58.6 (10.4)	
No	57.9 (7.6)	
Consulted with a dietitian/nutritionist		0.015
Yes	55.6 (10.8)	
No	61.5 (8.9)	

**Table 5:** Source of reference for sport nutrition information.

	*n* (%)		
	Very Unlikely/Unlikely	Neutral	Likely/Very Likely
Academician	1 (1.4)	12 (17.1)	57 (81.4)
Internet	5 (7.1)	21 (30.0)	44 (62.9)
Medical officer	10 (14.3)	20 (28.6)	40 (57.1)
Dietitian/nutritionist	11 (15.7)	21 (30.0)	38 (54.3)
Friend	12 (17.1)	25 (35.7)	33 (47.1)
Journal article	9 (12.9)	28 (40.0)	33 (47.1)
Coach/assistant coach	12 (17.1)	31 (44.3)	27 (38.6)
Parents	15 (21.4)	28 (40.0)	27 (38.6)
Magazine	13 (18.6)	32 (45.7)	25 (35.7)

**Table 6:** Preferred medium for nutrition education program.

Medium	*n*	%
Sports camp	51	33
Video	44	29
Talk	37	24
Brochure/poster	22	14

## Data Availability

The data presented in this study are available on request from the corresponding author. The data are not publicly available due to ethical.
